# Synthesis and Diels–Alder cycloaddition reaction of norbornadiene and benzonorbornadiene dimers

**DOI:** 10.3762/bjoc.5.39

**Published:** 2009-08-11

**Authors:** Bilal Nişancı, Erdin Dalkılıç, Murat Güney, Arif Daştan

**Affiliations:** 1 Atatürk University, Faculty of Science, Department of Chemistry, 25240 Erzurum-TURKEY

**Keywords:** benzonorbornadiene, Diels–Alder reaction, norbornadiene, Stille coupling

## Abstract

Dimeric forms of norbornadiene and benzonorbornadiene were synthesized starting with known monobromide derivatives. The Diels–Alder cycloaddition reaction of dimers with TCNE and PTAD was investigated and new norbornenoid polycyclics were obtained. All compounds were characterized properly using NMR spectroscopy.

## Introduction

Norbornadiene (**1**) and related compounds are of great scientific interest because of their unusual geometry and high reactivity. For example, these compounds exhibit a unique behavior in the cationic Wagner–Meerwein rearrangement [1–10], in the solvolytic reactivity [11], in the photochemical di-π-methane rearrangement [12–15], as well as in other instances [16–22]. Therefore, functionalizations of these compounds are important. In this study, we investigated the synthesis and Diels–Alder cycloaddition reaction of norbornadiene and benzonorbornadiene dimers.


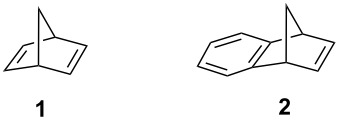


## Results and Discussion

**Scheme 1:**
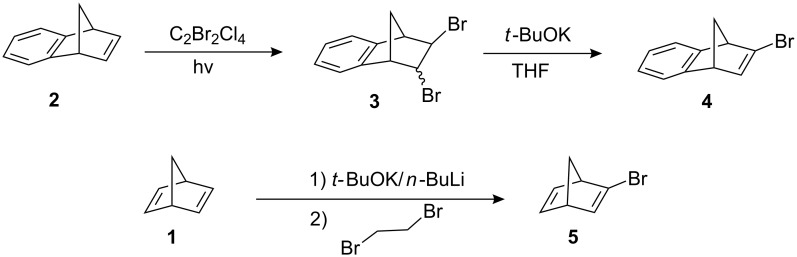
Synthesis of starting materials **4** and **5**.

One of the starting materials, 2-bromobenzonorbornadiene **4** was synthesized using a procedure described in the literature [15,23] (Scheme 1). Photochemical bromination of benzonorbornadiene **2** with 1,2-dibromotetrachloroethane gave isomeric dibromides **3** in high yield. Dehydrobromination reaction of dibromides **3** with potassium *tert*-butoxide resulted in the formation of monobromide **4**. The other starting material **5** was obtained using the reported procedures based on the use of potassium *tert*-butoxide/*n*-butyllithium super-base by starting with commercially available norbornadiene [24–27].

**Scheme 2:**
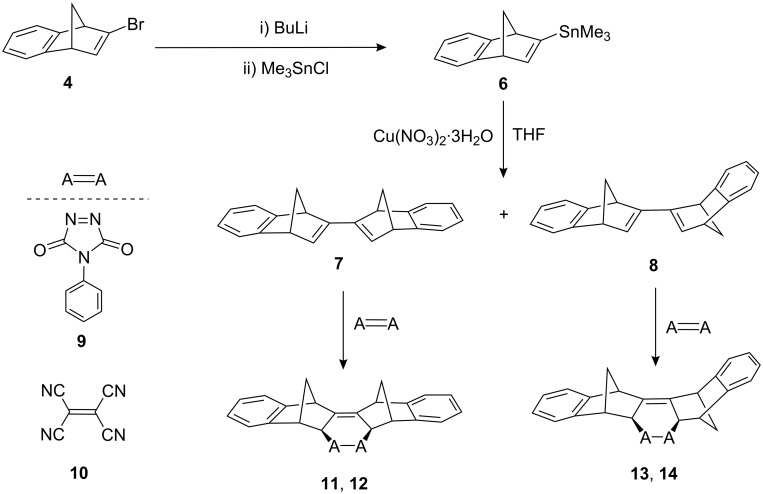
Synthesis and Diels–Alder cycloaddition reactions of dimers **7** and **8**.

When 2-bromobenzonorbornadiene **4** was treated with *n*-BuLi at −78 °C and the resulting anion was quenched with trimethyltin chloride, a single trimethyltin derivative **6** was observed in the crude reaction mixture and was finally isolated in 91% yield. Copper salts have been successfully employed for Stille-type hetero-coupling between unsaturated halides and stannanes [28–29]. Treatment of **6** with Cu(NO_3_)_2_·3H_2_O in dry THF at r.t. afforded the first synthesis of the expected dimers **7** and **8** in 25% yield in a 3:4 ratio, respectively, besides benzonorbornadiene **2** after column chromatography. The Diels–Alder cycloaddition of dimers **7** and **8** with PTAD (**9**) and TCNE (**10**) resulted in the formation of the corresponding products **11**–**14** in high yields (Scheme 2).

**Scheme 3:**
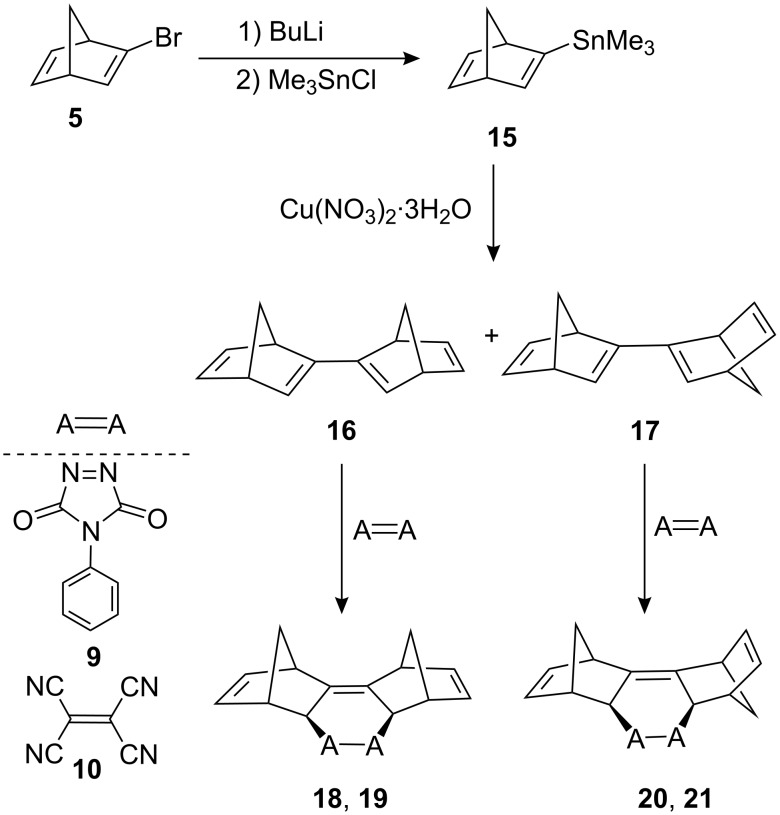
Synthesis and Diels–Alder cycloaddition reactions of dimers **16** and **17**.

Similarly, tin compound **15** was synthesized by the reaction of monobromide **5** with *n*-BuLi followed by reaction with trimethyltin chloride. Reaction of **15** with Cu(NO_3_)_2_·3H_2_O resulted in the formation of dimers **16** and **17** [30]. This reaction offered an alternative synthetic route to norbornadiene dimers **16** and **17**. The isomers **16** and **17** could not be separated, but after cycloaddition reaction of the mixture, the corresponding addition products **18**–**21** were isolated by chromatographic methods (Scheme 3).

### Structural Analyses

The determination of the structures of dimers **7**, **8** and dimers **16**, **17** by spectroscopic methods was not simple because the *C*_s_ symmetry of the *syn* dimers and the *C*_2_ symmetry of the *anti* dimers and the free rotation around the central σ bond make them indistinguishable. To determine which is which, cycloaddition reactions of dimers are more informative. Dimers **7** and **16** give symmetric addition products **11**, **12** and **18**, **19**, whereas the reaction of dimers **8** and **17** resulted in the formation of unsymmetrical products **13**, **14** and **20**, **21**.

**Figure 1:**
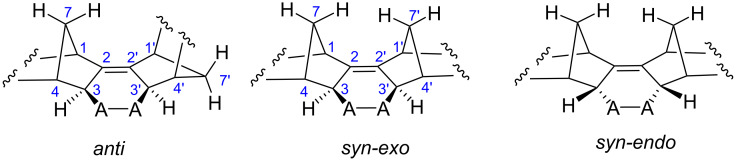
Numbering of carbon atoms and description of possible structures for dimers **11**–**14** and **18**–**21**.

For the symmetric addition products **11**, **12**, **18** and **19**, there are two possibilities: *exo* adduct or *endo* adduct (Figure 1). The coupling constants between the relevant protons in the norbornene unit are very informative to assign the correct configuration of the substituents [9–10]. The high value of *J*_34_ and *J*_3′4′_ (2.5–3.5 Hz) in the Diels–Alder addition products is uniquely accommodated by the *exo* orientation of the protons (*endo* orientation of -A-A- ring) at C^3^ and C^3′^ carbon atoms. For example, though there is coupling between the protons H^3^ and H^4^, there is no measurable coupling between the protons H_3′_ and H_4′_ in anti structures (Figure 1). On the other hand, the absence of any coupling between the related protons confirms the *endo* orientation of protons at C^3^ and C^3′^, which in turn proves the *exo*-orientation of the rings in adduct **11**, **12**, **18** and **19**. The coupling between the protons H^3^ (H^3′^) and H^7syn^ (H^7′syn^) (M or W orientation) also confirms the *exo* structures for **11**, **12**, **18** and **19** (Figure 1).

In summary, the synthesis and cycloaddition reaction of norbornadiene and benzonorbornadiene dimers was investigated and new norbornanoid polycyclic compounds, which open up several synthetic and mechanical investigations, were obtained.

## Experimental

*General:* Melting points are uncorrected. Infrared spectra were obtained from solution in 0.1 mm cells or KBr pellets on a regular instrument. The ^1^H and ^13^C NMR spectra were recorded on 400 (100) and 200 (50) MHz spectrometers. Apparent splitting is given in all cases. Column chromatography was performed on silica gel (60-mesh, Merck) TLC was carried out on Merck 0.2 mm silica gel 60 F_254_ analytical aluminum plates. All substances reported in this paper are *meso*-compounds or racemates.

**Synthesis of (1,4-dihydro-1,4-methano-naphthalen-2-yl)trimethylstannane (6):** A solution of *n*-BuLi in *n*-hexane (2.7 M, 3.41 mL, 9.19 mmol) was added dropwise to a solution of monobromobenzonorbornadiene **4** (2.03 g, 9.19 mmol) in dry THF (20 mL) at −78 °C and the resulting mixture was stirred for 40 min. Trimethyltin chloride (1.83 g, 9.19 mmol) was added portionwise and then left to warm to room temperature. The mixture was stirred overnight at room temperature. The crude product was washed with water (15 mL) and extracted with Et_2_O (2 × 50 mL) and then the combined ethereal extracts were dried over MgSO_4_ and concentrated in vacuo. (1,4-dihydro-1,4-methano-naphthalen-2-yl)trimethylstannane (**6**) was obtained as yellow liquid (2.55 g, 91%). ^1^H NMR (400 MHz, CDCl_3_): δ 7.26–7.20 (m, 2H, aryl), 7.06 (d, *J*_3,4_ = 2.9 Hz, 1H, H_3_), 6.98–6.94 (m, 2H, aryl), 4.08 (m, 1H, H_4_), 3.98 (m, 1H, H_1_), 2.24 (m, 2H, H_9_*_syn_* and H_9_*_anti_*), 0.18 (s, 9H, 3 × CH_3_). ^13^C NMR (100 MHz, CDCl_3_): δ 155.61, 153.22, 151.95, 151.92, 124.36, 124.18, 121.62, 121.58, 69.65, 55,77, 52.05, −9.70.

**Reaction of (1,4-dihydro-1,4-methano-naphthalen-2-yl)trimethylstannane (6) with Cu(NO_3_)_2_·3H_2_O:** Copper(II) nitrate trihydrate (345 mg, 1.4 mmol) was added portionwise to a solution of **6** (435 mg, 1.4 mmol) in THF (6 mL) at room temperature. The blue solution turned green within 1 h. The crude reaction mixture was diluted with Et_2_O (100 mL) and then washed with 5% NH_3_ (15 mL). The organic phase was dried over MgSO_4_ and concentrated in vacuo. The residue was chromatographed on neutral aluminum oxide (150 g) eluted with hexane. The first fraction was benzonorbornadiene (155 mg, 57%). The second fraction was *anti* isomer **8** (28 mg, 14%). ^1^H NMR (400 MHz, CDCl_3_): δ 7.15–6.79 (m, 8H, H^aryl^), 6.56 (d, *J*_3,4_ = *J*_3′,4′_ = 2.9 Hz, 2H, H^3^ and H^3′^), 3.92 (m, 2H, H^4^ and H^4′^), 3.86 (m, 2H, H^1^ and H^1′^), 2.40 (dt, A Part of AB system, *J*_9_*_syn_*_,9_*_anti_* = *J*_9′_*_syn_*_,9′_*_anti_* = 7.1 Hz, *J*_9_*_syn_*_,1_ = *J*_9_*_syn_*_,4_ = *J*_9′_*_syn_*_,1′_ = *J*_9′_*_syn_*_,4′_ = 1.5 Hz, 2H, H^9^*^syn^* and H^9′^*^syn^*), 2.25 (bd, B part of AB system, *J*_9_*_anti_*_,9_*_syn_* = *J*_9′_*_anti_*_,9′_*_syn_* = 7.1 Hz, 2H, H^9^*^anti^*^,9′^*^anti^*).^13^C NMR (100 MHz, CDCl_3_): δ 151.89, 151.46, 150.65, 133.96, 124.40, 124.32, 121.67, 120.97, 68.76, 52.12, 50.89. The third fraction was the *syn*-dimer **7** (23 mg, 11%). Colorless crystals from CH_2_Cl_2_/*n*-hexane (1:3). mp 152–154 °C. ^1^H NMR (400 MHz, CDCl_3_): δ 7.29–6.94 (m, 8H, H^aryl^), 6.61 (d, *J*_3,4_ = *J*_3′,4′_ = 2.9 Hz, 2H, H^3^ and H^3′^), 3.90 (m, 2H, H^4^ and H^4′^), 3.80 (m, 2H, H^1^ and H^1′^), 2.21 (dt, A Part of AB system, *J*_9_*_syn_*_,9_*_anti_* = *J*_9′_*_syn_*_,9′_*_anti_* = 7.3 Hz, *J*_9_*_syn_*_,1_ = *J*_9_*_syn_*_,4_ = *J*_9′_*_syn_*_,1′_ = *J*_9′_*_syn_*_,4′_ = 1.6 Hz, 2H, H^9^*^anti^* and H^9′^*^anti^*), 2.17 (bd, B Part of AB system, *J*_9_*_anti_*_,9_*_syn_* = *J*_9′_*_anti_*_,9′_*_syn_* = 7.3 Hz, 2H, H^9^*^anti^* and H^9′^*^anti^*). ^13^C NMR (100 MHz, CDCl_3_): δ 151.88, 151.55, 151.32, 134.38, 124.59, 124.41, 121.70, 121.15, 67.71, 51.59, 50.72. IR (KBr, cm^−1^): 3067, 2981, 2936, 2866, 1455, 1317, 1270, 1226, 1199, 1149, 1068, 1011, 909, 750, 735. MS (70 eV) *m/z*: 282.5 (M^+^, 32), 267.5 (21), 239.4 (5), 202.4 (2), 178.4 (5), 167.3 (26), 165.3 (32), 141.2 (28), 117.2 (71), 115.2 (56), 89.1 (6), 63.1 (3).

**Cycloaddition reaction of the dimer 7 with PTAD (9):** A solution of the *syn* dimer **7** (40 mg, 0.14 mmol) and PTAD (25 mg, 0.14 mmol) in 4 mL of CH_2_Cl_2_ was stirred at room temperature for 30 min. The solvent was removed under reduced pressure. The crude product was purified by crystallization from CH_2_Cl_2_/*n*-hexane (3:1) to give *syn* cycloadduct **11** (55 mg, 89%). Yellow crystals, mp 182–184 °C. ^1^H NMR (400 MHz, CDCl_3_): δ 7.60–7.12 (m, 13H), 4.66 (s, 2H), 4.24 (s, 2H), 3.75 (d, *J* = 1.5 Hz, 2H), 2.21 (dq, A Part of AB system, *J* = 9.4 Hz, *J* = 1.5 Hz, 2H), 2.14 (dt, B Part of AB system, *J* = 9.4 Hz, *J* = 1.4 Hz, 2H). ^13^C NMR (100 MHz, CDCl_3_): δ 151.74, 144.59, 144.54, 132.02, 131.84, 129.27, 128.28, 127.38, 127.05, 126.04, 123.09, 121.94, 59.25, 48.73, 48.20, 47.18. IR (KBr, cm^−1^): 3048, 2976, 2941, 1762, 1702, 1600, 1502, 1439, 1419, 1343, 1265, 1140. MS (70 eV) *m/z*: 458.4 (M^+^, 3), 344.0 (5), 282.0 (10), 280.8 (7), 165.6 (24), 119.4 (43), 116.4 (100), 91.3 (43).

**Cycloaddition reaction of the dimer 7 with TCNE (10):** A solution of the *syn* dimer **7** (50 mg, 0.17 mmol) and TCNE (**10**, 23 mg, 0.17 mmol) in 5 mL of CH_2_Cl_2_ was stirred at room temperature for overnight. The solvent was removed under reduced pressure. The crude product was purified by crystallization from CH_2_Cl_2_/*n*-hexane (3:1) to give *syn* cycloadduct **12** (68 mg, 93%). White crystals, mp 230–232 °C. ^1^H NMR (400 MHz, CDCl_3_): δ 7.54–7.17 (m, 8H, H^aryl^), 4.22 (s, 2H), 3.86 (m, 2H), 2.48 (bd, A Part of AB system, 2H, *J* = 10.3 Hz), 2.45 (m, 2H), 2.22 (d, B Part of AB system, 2H, *J* = 10.3 Hz). ^13^C NMR (100 MHz, CDCl_3_): δ 145.93, 144.40, 133.14, 127.76, 127.32, 122.17, 122.06, 112.08, 110.87, 50.01, 48.54, 47.81, 46.73, 45.90. IR (KBr, cm^−1^): 3050, 2955, 2872, 2306, 2254, 2217, 1463, 1318, 1265, 1120, 1153, 1013, 981, 785, 704. MS (70 eV) *m/z*: 410.1 (M^+^, 100), 394.1 (10), 370.1 (37), 345.1 (35), 319.1 (27), 295 (27), 267.1 (45), 265.0 (27), 229.0 (17), 205.0 (32), 176.9 (22), 164.9 (4), 152.9 (22), 151.9 (30).

**Cycloaddition reaction of the dimer 8 with PTAD (9):** A solution of the *anti* dimer (40 mg, 0.14 mmol) and PTAD (25 mg, 0.14 mmol) in 4 mL of CH_2_Cl_2_ was stirred at room temperature for 30 min. The solvent was removed under reduced pressure. The crude product was purified by crystallization from ether/*n*-hexane (2:1) to give *anti* cycloadduct **13** (58 mg, 90%). Yellow crystals, mp 168–170 °C. ^1^H NMR (400 MHz, CDCl_3_): δ 7.57–7.00 (m, 13H, H^aryl^), 4.94 (m, 1H), 4.52 (d, *J* = 2.3 Hz, 1H), 4.38 (m, 1H), 4.09 (m, 1H), 3.34 (m, 1H), 2.40 (dt, A part of AB system, *J* = 7.7 Hz, *J* = 1.5 Hz, 1H), 2.36 (dt, B part of AB system, *J* = 7.7 Hz, *J* = 1.5 Hz, 1H), 1.43 (bd, A part of AB system, *J* = 10.7 Hz, 1H), 1.25 (m, 1H), 0.46 (bd, B part of AB system, *J* = 10.7 Hz, 1H). ^13^C NMR (100 MHz, CDCl_3_): δ 155.20, 154.38, 150.70, 147.28, 147.18, 145.68, 142.93, 129.50, 129.35, 128.80, 128.53, 127.91, 127.53, 125.93, 125.85, 125.62, 125.41, 123.17, 122.43, 121.92, 69.32, 63.91, 62.90, 50.90, 49.64, 49.53, 49.27, 45.43. IR (KBr, cm^−1^): 3065, 2961, 2923, 2851, 1718, 1497, 1412, 1262, 1135, 1091, 1023, 801. MS (70 eV) *m/z*: 410.1 (M^+^, 100), 394.1 (10), 370.1 (33), 345.1 (31), 319.1 (26), 267.1 (45), 205.0 (33), 164.9 (44), 151.9 (32).

**Cycloaddition reaction of** **the dimer 8 with TCNE (10):** A solution of the *anti* dimer **8** (40 mg, 0.14 mmol) and TCNE (**10**, 18 mg, 0.14 mmol) in 5 mL of CH_2_Cl_2_ was stirred at room temperature for overnight. The solvent was removed under reduced pressure. The crude product was purified by crystallization from CH_2_Cl_2_/*n*-hexane (3:1) to give *anti* cycloadduct **14** (53 mg, 91%). White crystals, mp 240 °C (dec). ^1^H NMR (400 MHz, CDCl_3_): δ 7.55–7.17 (m, 8H), 4.15 (m, 1H), 4.13 (m, 1H), 3.92 (dd, *J* = 3.5 Hz, *J* = 1.5 Hz, 1H), 3.74 (m, 1H), 3.41 (dd, *J* = 3.5 Hz, *J* = 1.5 Hz, 1H), 2.52 (m, 1H), 2.31 (dt, A part of AB system, *J* = 9.5 Hz, *J* = 1.5 Hz, 1H), 2.11 (dt, A part of AB system, *J* = 10.3 Hz, *J* = 1.5 Hz, 1H), 2.06 (dt, B part of AB system, *J* = 9.5 Hz, *J* = 1.5 Hz, 1H), 2.02 (bd, B part of AB system, *J* = 10.3 Hz, 1H). ^13^C NMR (100 MHz, CDCl_3_): δ 146.29, 145.79, 144.63, 139.70, 132.89, 132.55, 128.80, 127.61, 127.33, 127.30, 126.60, 122.18, 121.98, 120.73, 112.40, 112.33, 109.23, 108.83, 52.93, 50.29, 49.19, 47.86, 47.71, 47.49, 47.43, 46.79, 45.12, 44.55. IR (KBr, cm^−1^): 3049, 2989, 2956, 2923, 2851, 2241, 1906, 1459, 1366, 1262, 1012, 984. MS (70 eV) *m/z*: 410.1 (M^+^, 40), 345.1 (13), 295.1 (10), 252.0 (7), 205.0 (13), 164.9 (12), 127.9 (8), 117.0 (30), 114.9 (100).

**Synthesis of (bicyclo[2.2.1]hepta-2,5-dien-2-yl)trimethylstannane (15):** A solution of *n*-BuLi in *n*-hexane (2.5 M, 1.2 ml, 2.9 mmol) was added dropwise to a solution of 2-bromobicyclo[2.2.1]hepta-2,5-diene (**5**, 0.50 g, 2.9 mmol) in dry THF (5 mL) at −78 °C and the resulting mixture was stirred for 1 h. Trimethyltin chloride (582 mg, 2.9 mmol) was added portionwise and then left to warm to room temperature. The mixture was stirred over night at room temperature. The crude product was washed with water (50 mL) and extracted with Et_2_O (2 × 50 mL) and then the combined ethereal extracts were dried over MgSO_4_ and concentrated in vacuo. (Bicyclo[2.2.1]hepta-2,5-dien-2-yl)trimethylstannane (**15**) was obtained in the form of a yellow liquid (700 mg, 95%). ^1^H NMR (400 MHz, CDCl_3_): δ 7.02 (bd, *J*_3,4_ = 2.9 Hz, 1H, H^3^), 6.70 (m, 1H, H^5^ or H^6^), 6.65 (m, 1H, H^5^ or H^6^), 3.76 (m, 1H, H^1^ or H^4^) 3.63 (m, 1H, H^1^ or H^4^), 1.91 (m, 1H, H^7^*^syn^* or H^7^*^anti^*), 1.88 (m, 1H, H^7^*^syn^* or H^7^*^anti^*), 0.12 (s, 9H, 3 × CH_3_).^13^C NMR (100 MHz, CDCl_3_): δ 155.46, 154.28, 143.12, 143.07, 74.70, 55.77, 52.15, −9.90.

**Reaction of (bicyclo[2.2.1]hepta-2,5-dien-2-yl)trimethylstannane (15) with Cu(NO_3_)_2_·3H_2_O:** Copper(II) nitrate trihydrate (1.13 g, 4.69 mmol) was added portionwise to a solution of **15** (1.2 g, 4.69 mmol) in THF (10 mL) at room temperature. The blue solution turned green within 40 min. The crude reaction mixture was diluted with Et_2_O (100 mL) and then washed with 5% NH_3_ (15 mL). The organic phase was dried over MgSO_4_ and concentrated in vacuo. The *syn*-dimer **16** and *anti*-dimer **17** (in a 46:54 ratio) were obtained as a mixture (130 mg, 30%). The isomeric dimers **16** [30] and **17** [30] could not be separated and were used as the mixture for the following step.

**Cycloaddition reaction of *syn*-16 and *anti*-17 mixture with PTAD:** A solution of mixture of *syn*-**16** and *anti*-**17** (120 mg, 0,66 mmol) and PTAD (116 mg, 0,66 mmol) in 10 mL of CH_2_Cl_2_ was stirred at room temperature for 30 min. The solvent was removed under reduced pressure. The residue was chromatographed on silica gel (30 g) column eluted with EtOAc/*n*-hexane (1:9).

The first fraction was *anti*-cycloadduct **20** (89 mg, 70% based on *anti* dimer **17**). Yellowish crystals from CH_2_Cl_2_/*n*-hexane (2:1), mp: 174–176 °C. ^1^H NMR (400 MHz, CDCl_3_): δ 7.53–7.25 (m, 5H, H), 6.33–6.27 (m, 3H), 6.06 (dd, *J* = 5.5 Hz, *J* = 2.9 Hz, 1H), 4.33 (d, *J* = 3.6 Hz, 1H), 4.09 (m, 1H), 3.93 (m, 1H), 3.68 (d, *J* = 1.7, 1H), 3.54 (m, 2H), 1.88 (dt, A part of AB system, *J* = 9.2 Hz, *J* = 1.7 Hz, 1H), 1.77 (bd, A part of AB system, *J* = 8.8 Hz, 1H), 1.68 (bd, B part of AB system, *J* = 9.2 Hz, 1H), 1.54 (bd, B part of AB system, *J* = 8.8 Hz, 1H). ^13^C NMR (100 MHz, CDCl_3_): δ 151.07, 149.85, 136.64, 136.55, 135.80, 133.12, 131.90, 131.61, 131.34, 129.20, 128.05, 125.87, 58.26, 56.54, 48.04, 47.57, 46.72, 46.37, 45.59, 45.38. IR (KBr, cm^−1^): 3060, 2925, 2852, 1760, 1698, 1502, 1419, 1130, 1028, 721. MS (70 eV) *m/z*: 357.3 (M^+^, 19), 315.8 (16), 291.2 (93), 250.9 (21), 239.2 (41), 195.1 (22), 182.1 (82), 118.8 (90), 91.0 (77), 77.0 (44). The second fraction was *syn*-cycloadduct **18** (75 mg, 69% based on *syn* dimer **16**) Yellowish crystals from CH_2_Cl_2_/*n*-hexane (2:1), mp: 194–196 °C. ^1^H NMR (400 MHz, CDCl_3_): δ 7.54–7.34 (m, 5H, H_aryl_), 6.28 (m, 4H), 4.10 (m 2H), 3.73 (m, 2H), 3.64 (m, 2H), 1.84 (bd, A Part of AB system, *J* = 9.0 Hz, 2H), 1.69 (bd, B part of AB system, *J* = 9.0 Hz, 2H). ^13^C NMR (100 MHz, CDCl_3_): δ 151.47, 136.83, 135.82, 131.83, 131.05, 129.25, 128.19, 126.06, 57.94, 48.10, 46.20, 45.66. IR (KBr, cm^−1^): 3060, 2962, 2929, 2863, 1760, 1700, 1502, 1422, 1279, 1139, 761, 729. MS (70 eV) *m/z*: 357.4 (M^+^, 4), 316.4 (4), 291.3 (54), 280.1 (3), 252.0 (4), 239.0 (7), 210.3 (9), 182.1 (25), 165.1 (35), 144.0 (71), 120.0 (16), 115.0 (40), 102.0 (9), 90.7 (65), 66.1 (23).

**Cycloaddition reaction of *syn*-16 and *anti*-17 *mixture* with TCNE (10):** A solution of mixture of *syn***-16 and ***anti*-**17** (103 mg, 0.56 mmol) and TCNE (**10**, 72 mg, 0.56 mmol) in 10 mL of CH_2_Cl_2_ was stirred at room temperature overnight. The solvent was removed under reduced pressure. The residue was chromatographed on silica gel (30 g) eluted with EtOAc/*n*-hexane (1:32). The first fraction was *anti*-1,4,5,8,8a,10a-hexahydro-1,4:5,8-dimethanophenanthrene-9,9,10,10-tetracarbonitrile (**21**) (82 mg, 87% based on *anti* dimer **17**). Yellowish crystals from CH_2_Cl_2_/*n*-hexane (3:1), mp:160–162 °C. ^1^H NMR (400 MHz, CDCl_3_): δ 6.46–6.35 (m, 4H), 3.61 (m, 2H), 3.49 (m, 1H), 3.36–3.33 (m, 2H), 2.36 (m, 1H), 2.16 (d, A part of AB system, *J* = 9.9 Hz, 1H), 1.95 (d, B Part of AB system, *J* = 9.9 Hz, 1H), 1.74–1.71 (m, 2H). ^13^C NMR (100 MHz, CDCl_3_): δ 138.63, 138.37, 138.27, 132.47, 131.96, 130.76, 113.25, 112.03, 110.50, 110.47, 51.40, 51.32, 48.01, 46.74, 46.47, 46.43, 45.63, 45.62, 44.79, 44.75. IR (KBr, cm^−1^): 2934, 2896, 2868, 2352, 2093, 1457, 1235, 1043, 960. MS (70 eV) *m/z*: 310.1 (M^+^, 83), 295.0 (17), 282.1 (70), 268.0 (57), 243.0 (73), 229.0 (55), 218.1 (80), 203.0 (48), 190.0 (52), 179.0 (40), 167.0 (100), 151.9 (53). The second fraction was *syn*-1,4,5,8,8a,10a-hexahydro-1,4:5,8-dimethanophenanthrene-9,9,10,10-tetracarbonitrile (**19**) (75 mg, 93% based on *syn* dimer **16**). Colorless crystals from CH_2_Cl_2_/*n*-hexane (2:1), mp: 136–138 °C. ^1^H NMR (400 MHz, CDCl_3_): δ 6.43 (dd, *J* = 5.5 Hz, *J* = 3.3 Hz, 2H), 6.30 (dd, *J* = 5,5 Hz, *J* = 2.9 Hz, 2H), 3.66 (s, 2H), 3.36 (d, *J* = 1.3 Hz, 2H), 2.48 (d, *J* = 1.3 Hz, 2H), 2.10 (d, A Part of AB system, *J_i_* = 9.5 Hz, 2H), 1.89 (d, B Part of AB system, *J* = 9.5 Hz, 2H). ^13^C NMR (100 MHz, CDCl_3_): δ 138.25, 137.78, 132.26, 112.39, 110.79, 49.49, 47.90, 46.52, 45.95, 45.30. IR (KBr, cm^−1^): 3066, 2995, 2951, 2874, 2247, 1454, 1317, 1007, 727. MS (70 eV) *m/z*: 310.1 (M^+^, 35), 282.1 (24), 268.1 (25), 242.1 (27), 228.1 (25), 217.1 (35), 204.1 (18), 189.0 (24), 178.1 (19), 167.1 (38), 126.9 (20), 115.2 (30), 101.3 (14), 91.0 (18), 88.1 (22), 76.1 (18), 65.5 (100).
